# 403. Clinical Characteristics and Outcomes of Patients with COVID-19 Admitted to the Intensive Care Unit in the Dominican Republic

**DOI:** 10.1093/ofid/ofab466.604

**Published:** 2021-12-04

**Authors:** Rita Alexandra Rojas-Fermin, Ann Sanchez, Anel E Guzman, Edwin Germosen, Cesar Matos, Alfredo J Mena Lora

**Affiliations:** 1 Hospital General Plaza de la Salud, Santo Domingo, Dominican Republic; 2 Universidad Tecnologica de Santiago, Santo Domingo, Distrito Nacional, Dominican Republic; 3 University of Illinois at Chicago, Chicago, Illinois

## Abstract

**Background:**

The disease caused by SARS-CoV-2, COVID-19, has caused a global public health crisis. Reported mortality rates across the world vary by region, local population characteristics and healthcare systems. There is a paucity of data on COVID-19 in low and middle income countries (LMICs). Our objective is to describe the clinical characteristics of critically ill patients with COVID-19 in the Dominican Republic (DR)

**Methods:**

We performed a retrospective review of patients admitted to the intensive care unit (ICU) with severe COVID-19 from March to December 31, 2020, at a 295-bed tertiary teaching hospital in the DR. Clinical characteristics, demographics, comorbidities, management and outcomes were tabulated. Survival was categorized by age and comorbidities.

**Results:**

A total of 382 patients were admitted to the ICU. The median age was 64 (range 14-97) and 64.3% (246) were male. Hypertension, diabetes, and obesity were the most common risk factors (Table 1). Corticosteroids were used in 91.6% (350), tocilizumab in 63% (82), and remdesivir in 31.6% (31). Antibacterials were used in 99.2% (379) of patients in the ICU. All-cause mortality in the ICU was 35.3% (135). Mortality was higher in older age groups (Figure 1) and in patients with multiple coexisting comorbidities (Figure 2).

Table 1. Comorbidities of patients with COVID-19 admitted to the ICU

**Conclusion:**

Hypertension, obesity and diabetes were common in critically ill patients with COVID-19 in the DR. Corticosteroids and tocilizumab were commonly used. Antibacterials were used in >99% of patients admitted to the ICU and may signal a target for future antimicrobial stewardship. Higher mortality rates were present in older age groups and those with multiple comorbidities. Risk of death increased drastically after age 40 and was comparative to those in advanced age groups. In patients with 4 comorbidities and above, mortality was more than three times higher.

**Disclosures:**

**All Authors**: No reported disclosures

**Table:** 

Comorbidity	N (%)
Hypertension	288 (75.3%)
Obesity	184 (48.16%)
Diabetes	181 (47.38%)
Chronic kidney disease	91 (23%)
Coronary artery disease	47 (12%)
Cerebrovascular disease	36 (9.4%)
Chronic obstructive pulmonary disease	27 (7.06%)
Malignancy	29 (7.5%)
Thyroid disease	23 (6%)

**Figure:**
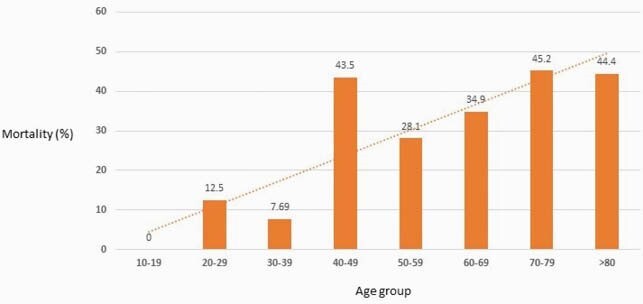
Figure 1. Mortality by age groups

**Figure:**
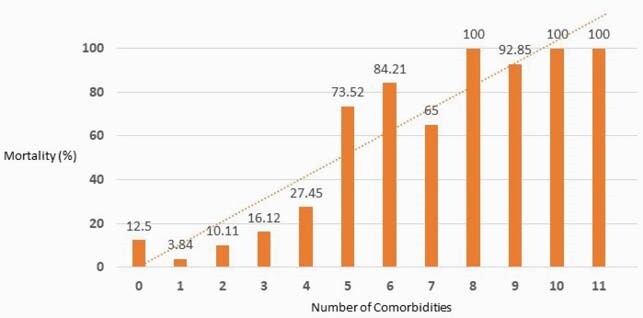
FIgure 2. Mortality by cumulative number of comorbidities

